# Ranking’s Abstract

**Published:** 1846-05-01

**Authors:** 


*Ranking's Abstract.*—The second part of the first volume of this work has lately been issued from the press of J. & H. G. Langley, Broadway, New York. This is a half-yearly publication, essentially on the same plan as Braithwaite's Retrospect, with which the profession are more familiar. It is intended to present a "practical and analytical digest of the contents of the principal British and Continental medical works published in the preceding six months;" and in addition to this, it embraces critical and elaborate reports on the progress of Midwifery, &c., Anatomy and Physiology, Chemistry, Forensic Medicine, and Materia Medica, by different writers, each eminent in his particular department. A copious Bibliographical Record is also appended. The work may be considered one of the cheapest of cheap publications in these days of cheap literature. The subscription price is $1.00 per annum. In advising our readers to buy it, we can conscientiously assure them that they will have *the worth of their money.*

